# Soft tissue healing in flapless versus conventional flap implant placement: A longitudinal study

**DOI:** 10.6026/973206300214000

**Published:** 2025-11-15

**Authors:** Rajaram Srinivasan, Rabia Khan, Adreet Hazra, Mamthashri Veeranjaneyalu, Dhruba Chatterjee, Abhishek Gaur

**Affiliations:** 1 Department of Dentistry, Pondicherry Institute of Medical Sciences, Ganathychettikulam, Pondicherry, India; 2 Department of Prosthodontics and Crown & Bridge, Sardar Patel Post Graduate Institute of Dental and Medical Sciences, Lucknow, Uttar Pradesh, India; 3 Department of Periodontology, Awadh Dental College and Hospital, Jamshedpur, Jharkhand, India; 4 Department of Oral and Maxillofacial Surgery, Government Dental College and Research Institute, Bengaluru, Karnataka, India; 5 Department of Oral and Maxillofacial Surgery, Naryana Health Barsat, Kolkata, West Bengal, India; 6 Department of Periodontology, Seema Dental College & Hospital, Rishikesh, Uttarakhand, India

**Keywords:** Aesthetic outcomes, gingival thickness, implant placement, soft tissue healing, surgical techniques

## Abstract

Soft tissue healing outcomes between flapless and conventional flap dental implant techniques in 310 patients is of interest. Data showed that flapless procedures resulted in faster healing times, greater gingival thickness, reduced pain and lower inflammation compared to the conventional flap technique. Both approaches had similar implant survival rates, but flapless implants had fewer post-operative complications. Patient satisfaction and aesthetic outcomes were significantly better for the flapless group. Thus, we show the clinical preference for flapless implant placement due to quicker recovery and fewer complications.

## Background:

Soft tissue healing plays a crucial role in the success of dental implant procedures, as it affects the stability, aesthetics and longevity of the implant [1]. The process of implant placement can be performed using two primary techniques: flapless implant placement and conventional flap surgery [2]. In flapless procedures, the implant is placed without the need for an incision in the gingiva, while conventional flap surgery involves making an incision to expose the underlying bone, providing direct visualization and access for implant placement [3]. Both techniques have distinct implications for soft tissue healing, which is influenced by factors such as the extent of trauma to the tissues, blood supply and the presence of peri-implant inflammation [4]. In flapless implant placement, the absence of an incision theoretically leads to fewer traumas to the surrounding soft tissues, promoting faster healing and minimizing the risk of postoperative complications [5].However, the lack of direct access to the bone may pose challenges in terms of implant positioning and achieving optimal soft tissue adaptation [6]. On the other hand, conventional flap surgery allows for greater control over the implant's positioning and the soft tissue envelope, but it may be associated with increased healing time and potential risks, such as wound dehiscence or infection, due to the surgical incision [7]. Therefore, it is of interest to describe the differences in soft tissue healing between flapless and conventional flap implant placement, as it can provide insights into which approach offers better outcomes in terms of tissue stability, aesthetics and overall patient recovery.

## Methodology:

This study was a prospective, randomized controlled trial (RCT) designed to evaluate and compare soft tissue healing outcomes between flapless and conventional flap implant placement techniques in a cohort of 310 patients. The patients were randomly divided into two groups, with 155 patients in each group. The study was conducted over a 12-month period to assess the healing process in both groups. Patients were selected based on specific inclusion and exclusion criteria. Inclusion criteria included patients aged 18-75 years who required single or multiple dental implants in an edentulous area, had adequate bone volume for implant placement, had no history of uncontrolled systemic diseases and were able to provide informed consent. Non-smokers or smokers who agreed to adhere to post-operative care instructions were also included. Exclusion criteria included patients with systemic health conditions that could interfere with healing (e.g., uncontrolled diabetes, autoimmune diseases), active periodontal disease or peri-implantitis, severe bone resorption requiring bone grafting and those who had undergone radiation therapy to the head and neck region. The 310 participants were randomly allocated into two groups: Group 1 (flapless technique) and Group 2 (conventional flap technique), with 155 patients in each group. Randomization was performed using computer-generated random numbers to ensure unbiased allocation. In the flapless technique group, implant placement was performed with minimal tissue manipulation, using a surgical guide for precise implant positioning and the punch technique or osteotomes to prepare the implant site without making any incisions in the gingiva. In contrast, patients in the conventional flap technique group underwent mucoperiosteal flap elevation to gain direct access to the bone, after which the implant was placed and the flap was repositioned and sutured to minimize trauma. Both groups received standardized post-operative care, which included antibiotic prophylaxis (e.g., amoxicillin) for 7 days following surgery, pain management (e.g., ibuprofen) and instructions on oral hygiene practices to avoid trauma to the surgical site. Follow-up visits were scheduled at 1 week, 1 month and 3 months after surgery to monitor healing progress. The primary outcome of the study was soft tissue healing, which was assessed at multiple time points. Key measures included healing time (the time required for soft tissue closure and complete mucosal healing), gingival thickness and height (measured using calipers or 3D scanning), pain and discomfort scores (assessed using a Visual Analog Scale at multiple time points) and soft tissue inflammation (evaluated using the modified Gingival Index at 1 week, 1 month and 3 months). Additionally, any postoperative complications such as infection, wound dehiscence, or delayed healing were recorded. Secondary outcomes included implant stability, patient satisfaction and aesthetic outcomes, which were assessed using a standardized aesthetic scale. Statistical analysis was conducted using SPSS software (version 25). Descriptive statistics summarized patient demographics and baseline characteristics. The healing outcomes between the two groups were compared using t-tests for normally distributed continuous variables, chi-square tests for categorical variables and ANOVA for comparing multiple time points. A p-value of less than 0.05 was considered statistically significant. The study was conducted in accordance with the Declaration of Helsinki and approved by the institutional review board (IRB) at the participating institution. Informed consent was obtained from all participants prior to enrollment. This methodology provided valuable insights into the differences in soft tissue healing between flapless and conventional flap implant placement techniques, contributing to the optimization of implant placement procedures and patient outcomes.

## Results:

The study assessed soft tissue healing outcomes following flapless and conventional flap implant placement techniques in 310 patients. The results are presented below, categorized into key outcomes such as healing time, gingival thickness, soft tissue inflammation, pain levels and complications. The demographic characteristics of the participants in both groups were similar, with no significant differences between the two groups in terms of age, gender, smoking status, or medical history. The average age of the patients was 45.6 years, with 60% male and 40% female. There were no statistically significant differences between the groups concerning baseline characteristics ( Table 1 - see PDF). The average healing time for the flapless technique was significant shorter compared to the conventional flap technique. In Group 1 (flapless), the average time for complete soft tissue healing was 4.2 weeks, while in Group 2 (conventional flap), it was 5.6 weeks (p < 0.01) (Table 2 - see PDF) Gingival thickness was significantly higher in Group 1 (flapless) at both 1 month and 3 months post-surgery. The mean gingival thickness in Group 1 was 2.8 mm at 1 month and 3.0 mm at 3 months. In Group 2, the thickness was 2.4 mm at 1 month and 2.6 mm at 3 months (p < 0.05) (Table 3 - see PDF). Pain levels were significantly lower in Group 1 (flapless) compared to Group 2 (conventional flap) at both the 1-week and 1-month follow-up. The average pain score in Group 1 was 3.2 at 1 week and 2.5 at 1 month. In Group 2, pain scores were 5.4 at 1 week and 4.2 at 1 month (p < 0.01) (Table 4 - see PDF). Soft tissue inflammation, assessed using the modified Gingival Index (GI), was significantly lower in Group 1 (flapless) at both 1 week and 1 month post-surgery. The average GI score in Group 1 was 1.2 at 1 week and 0.8 at 1 month. In Group 2, the GI score was 2.0 at 1 week and 1.6 at 1 month (p < 0.01) (Table 5 - see PDF). Post-operative complications were recorded in both groups. Group 1 (flapless) had fewer complications, with 6% of patients experiencing mild swelling, while Group 2 (conventional flap) had 12% of patients experiencing complications, including wound dehiscence and infection (p = 0.03) (Table 6 - see PDF). Implant stability was similar in both groups at the 3-month follow-up, with no significant differences between Group 1 (flapless) and Group 2 (conventional flap) in terms of implant success rates or stability (p = 0.45) (Table 7 - see PDF). Patient satisfaction scores, assessed using a Likert scale, were significantly higher in Group 1 (flapless). The average satisfaction score in Group 1 was 4.6, while in Group 2, it was 3.8 (p < 0.01) (Table 8 - see PDF). Aesthetic outcomes, as rated by an independent evaluator, were significantly better in Group 1 (flapless), with an average score of 4.5 compared to 3.7 in Group 2 (p < 0.01) (Table 9 - see PDF). Gingival recession was lower in Group 1 (flapless), with an average recession of 0.2 mm at 3 months, compared to 0.5 mm in Group 2 (p = 0.02) (Table 10 - see PDF).

## Discussion:

This study aimed to evaluate and compare soft tissue healing outcomes between flapless and conventional flap implant placement techniques in 310 patients. The results indicated that flapless implant placement was associated with quicker soft tissue healing, better gingival thickness, less pain and lower inflammation compared to the conventional flap technique. These findings align with several previous studies that have compared the two approaches, though there are also some differences in methodologies and conclusions. Below, we compare the findings of this study to five previous research articles that have examined soft tissue healing and clinical outcomes in flapless and conventional flap implant procedures. Jain *et al.* (2024) [8] conducted a study to compare flapless and conventional flap implant placements, focusing on soft tissue healing and post-operative complications. They found that flapless techniques resulted in faster soft tissue healing, with a significantly lower incidence of swelling and discomfort. This is consistent with the current study's findings, where patients in the flapless group experienced faster healing times (4.2 weeks vs. 5.6 weeks for conventional flap). However, Das (2023) *et al.* [9] noted that flapless procedures posed a higher risk of implant misplacement due to the lack of direct visualization, a factor that was not significantly problematic in this study due to the use of surgical guides. Therefore, this study provides additional evidence of the benefits of flapless procedures for faster healing while mitigating some of the earlier concerns. Gao *et al.* (2021) [10] explored the impact of flapless versus conventional flap techniques on gingival contour and with the current study, where patients aesthetics, reporting that flapless surgery led to better aesthetic outcomes in terms of both soft tissue volume and contour. This aligns in the flapless group showed better gingival thickness and height, as measured at both 1 and 3 months post-surgery (2.8 mm vs. 2.4 mm at 1 month). Additionally, patients in the flapless group reported higher satisfaction scores and better aesthetic outcomes, which corroborates Chandrshekhar *et al.* (2024) [11] conclusion that flapless implant placement tends to preserve the natural gingival contour and provides more favorable aesthetic results. Divakar *et al.* (2020) [12] focused on the pain and discomfort associated with flapless and conventional flap procedures. Their study found that patients who underwent flapless surgery reported significantly less pain and discomfort compared to those who had conventional flap surgery, particularly in the first week post-surgery. This study is in agreement with the current study, where pain scores at 1 week were significantly lower in the flapless group (3.2 vs. 5.4) and continued to show lower pain levels at 1 month (2.5 vs. 4.2). The present study builds upon Fortin *et al.*'s [13] findings, suggesting that flapless implant placement not only reduces pain but also enhances post-operative recovery, leading to greater patient satisfaction. Cai *et al.* (2020) [14] compared implant survival and soft tissue healing outcomes between flapless and conventional flap techniques. Their results showed that both approaches had similar implant survival rates, but flapless procedures resulted in faster healing and fewer soft tissue complications. This aligns with the findings of the current study, where implant survival was similar between the groups (98% in Group 1 and 97% in Group 2) and flapless techniques led to significantly faster soft tissue healing. The current study supports assertion that while implant survival rates may not differ significantly, flapless procedures offer faster recovery and fewer complications, providing an overall advantage in clinical practice.

## Conclusion:

In conclusion, this study demonstrates that flapless implant placement results in faster soft tissue healing reduced pain and better aesthetic outcomes compared to conventional flap techniques. Both techniques show similar implant survival rates, but flapless procedures offer quicker recovery and fewer complications. Thus, we show the growing preference for flapless implant placement in clinical practice.
